# Prevalence of thyroid dysfunction in patients with acute atrial fibrillation attended at a cardiology emergency room

**DOI:** 10.1590/S1516-31802003000400004

**Published:** 2003-07-01

**Authors:** Juarez Neuhaus Barbisan, Flávio Danni Fuchs, Beatriz D'Agord Schaan

**Keywords:** Atrial fibrillation, Thyroid dysfunction, Hyperthyroidism, Fibrilação atrial, Tireóide, Hipertireoidismo. Hormônio T_3_ da tireóide, Hormônio T_4_ da tireóide

## Abstract

**CONTEXT:**

Atrial fibrillation occurs frequently in patients with thyrotoxicosis, while it has low prevalence in adults of the general population. The prevalence of thyroid dysfunction in subjects with atrial fibrillation is 0 to 24%, a wide variation that is attributed to the different methodologies applied. However, continuous use of amiodarone in patients with previous atrial fibrillation may interfere with these prevalence rates.

**OBJECTIVE:**

In this study, we present the prevalence of thyroid dysfunction in adult patients who presented at a cardiac emergency room with acute atrial fibrillation, using a sensitive thyroid-stimulating hormone (TSH) assay and triiodothyronine (T_3_) and thyroxine (T_4_) determination.

**TYPE OF STUDY:**

Cross-sectional study

**SETTING:**

Emergency room of a tertiary care facility.

**PARTICIPANTS:**

A total of 72 patients with atrial fibrillation who presented at the emergency room not more than 48 hours after its onset.

**PROCEDURES:**

A standardized questionnaire and 12-lead electrocardiogram were applied, and T3, T4 and TSH were determined.

**MAIN MEASUREMENTS:**

TSH, T_3_ and T_4_ determination.

**RESULTS:**

Among these patients, 16.6% had altered thyroid function tests: 6.9% had hyperthyroidism, 5.6% hypothyroidism and 4.2% had increased T4 levels, by means of amiodarone use.

**CONCLUSION:**

The high prevalence of thyroid dysfunction in our study, especially hyperthyroidism, suggests that routine thyroid testing with sensitive thyroid-stimulating hormone assay is required in patients with acute atrial fibrillation.

## RESUMO

**CONTEXTO:**

A fibrilação atrial ocorre freqüentemente em pacientes com hipertireoidismo, enquanto que, na população adulta em geral, sua prevalência é muito baixa. A prevalência de disfunção tireoidiana em indivíduos com fibrilação atrial varia de 0% a 24%; esta grande variação sendo atribuída às diferentes metodologias aplicadas em seu diagnóstico.

**OBJETIVO:**

Neste estudo, apresentamos a prevalência de disfunção tireoidiana em pacientes adultos que se consultaram no pronto socorro cardiológico com fibrilação atrial aguda, utilizando, para seu diagnóstico, as determinações séricas de tireotrofina ultrassensível, triiodotironina (T_3_) e tiroxina (T_4_).

**TIPO DE ESTUDO:**

Estudo transversal.

**LOCAL:**

Emergência do Instituto de Cardiologia do Rio Grande do Sul.

**PARTICIPANTES:**

72 pacientes com fibrilação atrial de até 48 horas de duração.

**PROCEDIMENTOS:**

Os participantes foram submetidos a questionário, eletrocardiograma e tiveram coletada amostra de sangue.

**VÁRIÁVEIS ESTUDADAS:**

Tireotrofina sérica, T_3_ e T_4_.

**RESULTADOS:**

Dos pacientes estudados, 16,6% tinham alteração de prova de função tireoidiana: 6,9% apresentavam hipertireoidismo, 5,6% apresentavama hipotireoidismo e 4,2% tinham apenas elevação dos níveis de T_4_ pelo uso de amiodarona.

**CONCLUSÕES:**

A alta prevalência de disfunção tireoidiana observada em nosso estudo, especialmente de hipertireoidismo, sugere que testes de função tireoidiana sejam realizados rotineiramente em todos os pacientes que se apresentem com fibrilação atrial aguda.

## Introduction

Atrial fibrillation occurs in 9 to 22% of patients with hyperthyroidism, contrasting with a prevalence of 0.4 to 4.0% in adults of the general population.^1,2^ In addition to its disabling symptoms, atrial fibrillation is a strong risk factor for systemic embolism, especially with regard to cerebral circulation.^3^

Estimates of thyroid dysfunction prevalence in subjects with atrial fibrillation range from 0 to 24%.^4,5^ This large variation may arise from different ages of subjects evaluated,^6,7^ different regions where the studies were carried out,^8^ presence of associated illness and use of medications^9^ and different methods of measuring thyroid hormones and thyroid-stimulating hormone.^10,11^

The development of thyroid-stimulating hormone assays with improved sensitivity has changed the diagnosis of thyroid dysfunction. Subclinical hypo and hyperthyroidism are being diagnosed by the presence of high and low thyroid-stimulating hormone, respectively, in association with normal free thyroid hormone values in asymptomatic individuals.^12^ The pathophysiological importance of these new categories of thyroid dysfunction has been highlighted by new studies that have demonstrated that both subclinical hypo and hyperthyroidism are potentially reversible with treatment.^13-15^ Sawin et al., in a sample of 2,007 individuals of more than 60 years of age, found a threefold greater risk of atrial fibrillation in the presence of low serum thyrotropin concentrations caused by endogenous or exogenous subclinical thyrotoxicosis.^11^

The prevalence of hyperthyroidism in individuals presenting with atrial fibrillation has been evaluated in a few studies using the diagnostic criterion of suppressed thyroid-stimulating hormone, as measured by a sensitive assay. These studies only diagnosed hyperthyroidism in individuals who had elevated serum thyroxine levels in addition to suppressed thyroid-stimulating hormone. They did not take into account that subclinical hyperthyroidism may be accompanied by cardiovascular dysfunction.^4,16,17^

In this study, we present the prevalence of thyroid dysfunction in adult patients with acute atrial fibrillation who were attended at a cardiac emergency room, using a sensitive thyroid-stimulating hormone assay and triiodothyronine (T_3_) and thyroxine (T_4_) determination.

## Methods

From June 1997 to October 1999, a total of 72 patients who presented at the emergency room of our Institution with atrial fibrillation, no more than 48 hours after its onset, were investigated. All these patients agreed to participate in a clinical trial to test the efficacy of procainamide.^18^ All patients answered a brief questionnaire that included identification data, age, medications in use, medical history and thyroid disease history. The patients included could have chronic ischemic heart disease or another structural heart disease but were not to present acute manifestation (myocardial infarction, for example) or hemodynamic instability. A history of thyroid disease was not an exclusion criterion.

They underwent clinical examination and electrocardiogram, and blood samples for thyroid function tests, investigating the thyroid hormones triiodothyronine (T_3_) and thyroxine (T_4_) and sensitive thyroid-stimulating hormone (TSH). The diagnosis of thyroid dysfunction was based exclusively on hormonal determinations, since diagnoses based on clinical grounds have been shown to have low sensitivity and specificity.^16^ The criterion used for diagnosing hyperthyroidism was based on the guidelines of the American Thyroid Association. These suggest that virtually all types of hyperthyroidism encountered in clinical practice should be accompanied by serum sensitive thyroid-stimulating hormone concentrations of less than 0.1 mIU/l and not levels that are just below the normal range.^19^ On the other hand, high levels of sensitive thyroid-stimulating hormone, even if just above the upper limit of the normal range, are implicated in depression and higher levels of cholesterol, which are representative of tissue hypothyroidism.^20^

T_3_, T_4_ and TSH were measured by polarized fluorescence using commercial kits (Abbott, Park, IL, USA). The reference value for T_3_ was 70-231 ng/dl, T_4_ 4.5-12.5 ng/dl and sensitive thyroid-stimulating hormone 0.32-5.0 mU/l. Patients with sensitive thyroid-stimulating hormone less than or equal to 0.1 mU/l were considered to have hyperthyroidism, which was subclinical if T_3_ and T_4_ were normal and clinical if T_3_ and/or T_4_ were high. Patients with sensitive thyroid-stimulating hormone greater than or equal to 5 mU/l were considered to have hypothyroidism, which was subclinical if T_3_ and T_4_ were normal and clinical if T_3_ and/or T_4_ were low. High levels of thyroxine, in association with normal levels of sensitive thyroid-stimulating hormone in patients using amiodarone, were considered an adverse drug effect.

## RESULTS

Table 1 presents the ages and numbers of patients with hypothyroidism, hyperthyroidism or altered thyroid function tests attributed to amiodarone use, according to sex. Among the 72 patients who presented with acute atrial fibrillation at the cardiology emergency room of the Cardiology Institute, 16.6% had altered thyroid function tests: five patients had hyperthyroidism, four had hypothyroidism and only three had high T_4_, with normal sensitive thyroid-stimulating hormone. These latter three cases were attributed to the use of amiodarone, for which the most common side effect regarding thyroid function is an increase in T_4_ levels. Of the five hyperthyroid patients, two were using amiodarone, and of the four hypothyroid patients, one was using this drug. Among the patients with no thyroid function test abnormality (60 patients), only three were using amiodarone. Among the patients with hyperthyroidism, four out of five were women; and among the four with hypothyroidism, three were women.

**Table 1:** Clinical characteristics of all patients studied

	Men	Women	Total
Number	37	35	**72**
Age (years)	â51.9 ± 14.3	â59.3 ± 11.2	**55.5 ± 13.3**
Structural Cardiopathy, n (%)	â22 (59.5)	â17 (48.6)	**35 (48.6)**
Cardiovascular Drugs, n (%)	â17 (45.9)	â16 (45.7)	**33 (44.4)**
Hyperthyroidism, n (%)	â1 (1.4)	â4 (5.6)	**5 (6.9)**
Hypothyroidism, n (%)	â1 (1.4)	â3 (4.2)	**4 (5.6)**
Dysfunction due to amiodarone, n (%)	â1 (1.4)	â2 (2.8)	**3 (4.2)**

The overall prevalence of thyroid dysfunction, including the clinical and subclinical cases, was 12.5%. Table 2 presents the individual data for each patient with thyroid dysfunction. Amiodarone was being used by six patients. Two of them had hyperthyroidism, one had hypothyroidism and three had high T_4_ with normal sensitive thyroid-stimulating hormone, thus characterizing an adverse drug effect (Tables 1 and 2).

**Table 2:** Clinical characteristics of patients presenting with thyroid dysfunction

Case no.	Gender	Age	T_3_(ng/dl)	T_4_(ng/dl)	Thyroid-stimulating hormone (mU/l)	Final diagnosis
1	M	51	117	14.9	1.52	Amio
2	F	61	144	14.2	0.03	Hyper/Amio
3	M	53	102	18.2	0.1	Hyper/Amio
4	F	60	80	14.3	1.3	Amio
5	F	50	254	17	0.02	Hyper
6	F	24	206	12.8	1.54	Amio
7	F	65	177	18.8	0.02	Hyper
8	F	59	108	4.5	11.2	Hypo
9	F	72	99	-	26	Hypo/S/Amio
10	F	55	120	7.4	8.3	Hypo/S
11	M	51	104	8.5	22.4	Hypo/S
12	F	62	89	10.1	0.07	Hyper/S

*F: female; M: male; Hyper: Hyperthyroidism; Hypo: Hypothyroidism; Amio: amiodarone; S: Subclinical*

## DISCUSSION

This study demonstrated a high prevalence of thyroid dysfunction in a large sample of patients with acute atrial fibrillation attended at a cardiac emergency room, independent of the use of amiodarone. Both hypo and hyperthyroidism were frequent, but only the latter exceeded what was expected for the general population at the same age as our patients.

Previous studies have shown widely varying estimates of thyroid dysfunction incidence in patients with atrial fibrillation, probably resulting from the use of different methods for determining thyroid hormone levels, variation in diagnostic limits, and evaluation of different populations.^5,7,21^ Forfar et al.^21^ and Ciaccheri et al.^22^ described thyrotoxicosis prevalence of around 13%. Their data, however, were based on a flat thyroliberin test, which presents many limitations for diagnosing hyperthyroidism. There is no general agreement about the lower normal limit for this test, since an absence of response may be attributed to the variable secretion of thyroid-stimulating hormone.^16^ A low response to thyroliberin may also be found under several other conditions, such as psychiatric diseases, acute diseases, diabetes mellitus, lower food intake, heart failure, renal failure, treatment with certain drugs (corticosteroids, verapamil, aspirin and oral contraceptives), old age and others.^23^ Moreover, Davies et al. demonstrated that elderly patients with atrial fibrillation who showed no thyroid-stimulating hormone response to thyrotropin-releasing hormone at the beginning of their study recovered their response to thyrotropin-releasing hormone six weeks later.^24^

With the availability of the new generation of sensitive thyroid-stimulating hormone immunoassays, the thyroliberin test has become regarded as redundant. Current second and third generation assays have detection limits of 0.05 and 0.005 mU/l, respectively. Serum sensitive thyroid-stimulating hormone concentration was undetectable in patients with nonthyroid illness when a second-generation assay was used and in all patients when a third-generation assay was used, whereas virtually all patients with thyrotoxicosis had undetectable levels with both assays.^25^ A finding of undetectable sensitive thyroid-stimulating hormone from such assays is a reliable indicator of suppressed thyroid-stimulating hormone secretion, which indicates the presence of hyperthyroidism, especially in a sample of patients with atrial fibrillation. However, no standard numerical value has been assigned to the serum concentration of sensitive thyroid-stimulating hormone below which suppression is considered to occur. The limits vary from center to center, depending on the sensitivity of the local assay. Recently, guidelines from the American Thyroid Association for the detection of thyroid dysfunction have suggested that sensitive thyroid-stimulating hormone of less than 0.1 mIU/l should be the diagnosis for hyperthyroidism. This was therefore the limit employed in our analysis.^19^

Two other studies that have used sensitive thyroid-stimulating hormone for diagnosing hyperthyroidism found prevalences of 2.7%^16^ and 0.7%.,^17^ which were much lower than our results. The first of these, however, did not use sensitive thyroid-stimulating hormone measurement alone for diagnosing hyperthyroidism, but required suppressed sensitive thyroid-stimulating hormone with high levels of thyroid hormones or a flat thyroliberin test. This approach does not consider the existence of subclinical thyrotoxicosis, a condition that is defined by normal free T_4_ and T_3_ serum concentrations accompanied by undetectable sensitive thyroid-stimulating hormone concentration, in patients who are not chronically ill, irrespective of the presence of symptoms attributed to thyroid dysfunction.^1^ The disparity of our data with those found by Krahn et al.,^17^ who employed the same criteria for diagnosing hyperthyroidism, may be ascribed to regional differences in the prevalence of thyroid disease.

Minor degrees of thyroid dysfunction that seem inconsequential in an endocrinological setting may be important in patients who are particularly vulnerable to changes in thyroid function. Subclinical thyrotoxicosis that is spontaneous, or results from the use of thyroid-stimulating hormone-suppressive doses of levothyroxine for thyroid carcinoma, nontoxic goiter or hypothyroidism, has clear pathophysiological consequences, especially regarding bone metabolism and cardiac function.^26^ Individuals with subclinical hyperthyroidism due to levothyroxine who were studied using Doppler echocardiography and 24-hour Holter electrocardiogram monitoring presented an increase in 24-hour mean heart rate and supraventricular arrhythmia, as well as increased left ventricular mass index, higher rates of systolic function and impairment of diastolic function.^13^ When patients with subclinical thyrotoxicosis were treated with antithyroid drugs for six months, there was a fall in the number of premature ectopic atrial and ventricular beats, and a decrease in left ventricular mass.^15^ These findings, with the demonstration that suppressed sensitive thyroid-stimulating hormone is predictive of a higher incidence of atrial fibrillation,^11^ make it clear that suppressed sensitive thyroid-stimulating hormone identifies hyperthyroidism in patients with atrial fibrillation. This may be different from the general population, where reduced sensitive thyroid-stimulating hormone concentration may be secondary to hypopituitarism.^27^

We did not exclude patients using medications that could interfere with thyroid function or those with a previous history of thyroid diseases, as other authors have done.^23^ But we did not include the three patients using amiodarone, with high T4 levels without suppressed sensitive thyroid-stimulating hormone, among the prevalent cases of hyperthyroidism. The exclusion of patients with a previous diagnosis of thyroid dysfunction would bias the estimates of prevalence of hyperthyroidism among patients with acute atrial fibrillation.

The 5.6% prevalence of hypothyroidism that we observed only reflects the prevalence of this disease in the general population, which is estimated to be between 2.3 and 10.3%.^28,29^ On the other hand, the prevalence of hyperthyroidism in the general population ranges from 0.5 to 2.5%,^1,29,30^ and thus is much lower than our findings, which underscores the possibility that hyperthyroidism is a cause or a trigger of atrial fibrillation in some patients. High prevalence of hyperthyroidism is characteristic of iodine-deficient regions, but this seems unlikely in our cases, because the Brazilian program of salt iodination has attained its goals over the last decade.^31^ We must emphasize that we have not studied a control group: this would ideally be represented by patients coming to the cardiology emergency room with other complaints and presenting sinus rhythm on the electrocardiogram, which would allow statistical confirmation of our hypothesis.

## CONCLUSION

The high prevalence of thyroid dysfunction in our study, especially hyperthyroidism, suggests that routine thyroid testing using sensitive thyroid-stimulating hormone assay is required in patients with acute atrial fibrillation.
